# A systematic review and meta-analysis of conservative management of Achilles tendinopathy

**DOI:** 10.1186/1757-1146-4-S1-P32

**Published:** 2011-05-20

**Authors:** Samuel P Leitch, Andrea E Bialocerkowski, Stuart J Warden, Natalie J Collins, Andy W Chien, Kay M Crossley

**Affiliations:** 1 Department of Physiotherapy, The University of Melbourne, Melbourne, 3010, Victoria, Australia; 2 Department of Physiotherapy, University of Western Sydney, School of Biomedical and Health Sciences, Campbelltown, 2560, New South Wales, Australia; 3 Department of Physical Therapy, School of Health and Rehabilitation Sciences, Indiana University, Indianapolis, 46236, USA; 4 Department of Mechanical Engineering, The University of Melbourne, Melbourne, 3010, Victoria, Australia

## Background

This systematic review and meta-analysis was conducted to provide a clear guide for the best evidence based approach to conservatively manage Achilles tendinopathy. A recent systematic review provided a descriptive summary of the evidence with the absence of a quality assessment of the study methodologies.

## Methods

A systematic literature search was conducted to identify randomised, controlled studies using pain or function outcome measures which were published in English. Quality assessments were performed using the Modified PEDro rating scale. Standardised mean differences for each intervention and pooled data were calculated using Review Manager software.

## Results

The search strategy yielded 22 studies whose methodological quality rated between 2 and 12 out of 14 on a Modified PEDro rating scale. Studies were grouped by their primary intervention (eccentric exercise, shock wave therapy, ultrasound, night splints and other conservative management options). Follow up times were predominantly within three months. A meta-analysis was able to be performed for two intervention comparisons; shock wave therapy (SWT) versus eccentric exercise (EE) and laser therapy versus a sham laser therapy, where both groups received an EE program. The pooled data found a moderate significant effect favouring SWT and small significant effect favouring laser therapy. Of the eleven studies evaluating EE, six reported that EE had superior results to the control intervention.

## Conclusions

This systematic review emphasises the need for a consistent use of valid and reliable outcome measures, larger subject numbers and longer follow-up times to build a larger body of high quality evidence and a greater opportunity to perform meta-analyses using studies that examine conservative interventions for Achilles tendinopathy. The current evidence supports the use of EE, SWT and laser therapy for the management of Achilles tendinopathy.

